# Marginal Zone Lymphoma Complicated by Protein Losing Enteropathy

**DOI:** 10.1155/2016/9351408

**Published:** 2016-11-09

**Authors:** Nadine Stanek, Peter Bauerfeind, Guido Herzog, Henriette Heinrich, Matthias Sauter, Daniela Lenggenhager, Cäcilia Reiner, Markus G. Manz, Jeroen S. Goede, Benjamin Misselwitz

**Affiliations:** ^1^Department of Gastroenterology and Hepatology, University Hospital Zurich, Zurich University, Zurich, Switzerland; ^2^Department of Gastroenterology, Stadtspital Triemli Zurich and Zurich University, Zurich, Switzerland; ^3^Department of Pathology and Molecular Pathology, University Hospital Zurich, Zurich University, Zurich, Switzerland; ^4^Department of Radiology, University Hospital Zurich, Zurich University, Zurich, Switzerland; ^5^Department of Hematology, University Hospital Zurich, Zurich University, Zurich, Switzerland

## Abstract

Protein losing enteropathy (PLE) refers to excessive intestinal protein loss, resulting in hypoalbuminemia. Underlying pathologies include conditions leading to either reduced intestinal barrier or lymphatic congestion. We describe the case of a patient with long-lasting diffuse abdominal problems and PLE. Repetitive endoscopies were normal with only minimal lymphangiectasia in biopsies. Further evaluations revealed an indolent marginal zone lymphoma with minor bone marrow infiltration. Monotherapy with rituximab decreased bone marrow infiltration of the lymphoma but did not relieve PLE. Additional treatments with steroids, octreotide, a diet devoid of long-chain fatty-acids, and parenteral nutrition did not prevent further clinical deterioration with marked weight loss (23 kg), further reduction in albumin concentrations (nadir 8 g/L), and a pronounced drop in performance status. Finally, immunochemotherapy with rituximab and bendamustine resulted in hematological remission and remarkable clinical improvement. 18 months after therapy the patient remains free of gastrointestinal complaints and has regained his body weight with normal albumin levels. We demonstrate a case of PLE secondary to indolent marginal zone lymphoma. No intestinal pathologies were detected, contrasting a severe and almost lethal clinical course. Immunochemotherapy relieved lymphoma and PLE, suggesting that a high suspicion of lymphoma is warranted in otherwise unexplained cases of PLE.

## 1. Introduction

Protein losing enteropathy (PLE) comprises excessive loss of proteins into the gastrointestinal tract resulting in hypoproteinemia. A variety of conditions can result in intestinal leakage via two mechanisms [1]: (i) reduced intestinal barrier due to mucosal injury and (ii) lymphatic congestion and/or stasis resulting in increased lymphatic pressure.

Therefore, work-up of PLE includes testing for malignancy, celiac disease, inflammatory bowel-diseases, chronic infectious diseases, collagenous colitis, allergic gastroenteropathy, eosinophilic gastroenteritis, and amyloidosis [1–10]. Ménétrier's disease characterized by giant mucosal folds in the stomach might be a common cause of PLE.

Lymphatic congestion or stasis can result in leakage of intestinal lymph into the lumen of intestinal organs. Diagnostic clues for increased lymphatic pressure include dilated lymphatic vessels (lymphangiectasia) on histological specimen. Intestinal lymphangiectasia has been described as a primary condition or as a complication of other diseases [11]. Secondary causes for increased lymphatic pressure include cardiac disease, portal hypertension, damage or injury of retroperitoneal lymph nodes, for instance, after surgery, chemotherapy or infectious or malignant conditions, or Turner's syndrome [12].

We report the case of severe paraneoplastic PLE in a patient with marginal zone lymphoma but without gastrointestinal lesions. Immunochemotherapy resulted in remission of lymphoma and relief of all symptoms.

## 2. Case Presentation 

A 47-year-old patient was referred to our outpatient clinic for evaluation of abdominal complaints since 15 years. The patient described postprandial nausea with diffuse abdominal pain and diarrhea hours after the meal. Five years ago the patient noted ankle edema and hypoalbuminemia was diagnosed.

The patient was a sportive nonsmoker. Medication included pantoprazole and vitamin supplements. Besides appendectomy the personal history was unremarkable. Physical examination revealed mild peripheral edema but no further findings.

Blood work was remarkable for hypoalbuminemia 18 g/L (norm 40–49 g/L) and mild iron deficiency. Liver and kidney function tests and a peripheral blood count were normal (Table 1). In 24 h stool measurements alpha-1 antitrypsin clearance was 432 mL/d (norm <24 mL/d), confirming protein losing enteropathy. Repetitive stool tests revealed normal calprotectin levels and no evidence for bacterial or parasitic infection. An HIV test was negative.

Chromogranin A and Gastrin were repeatedly elevated, most likely due to pantoprazole treatment and ^68^Ga DOTATATE-PET-scan did not reveal a neuroendocrine tumor.

**Figure 1:**
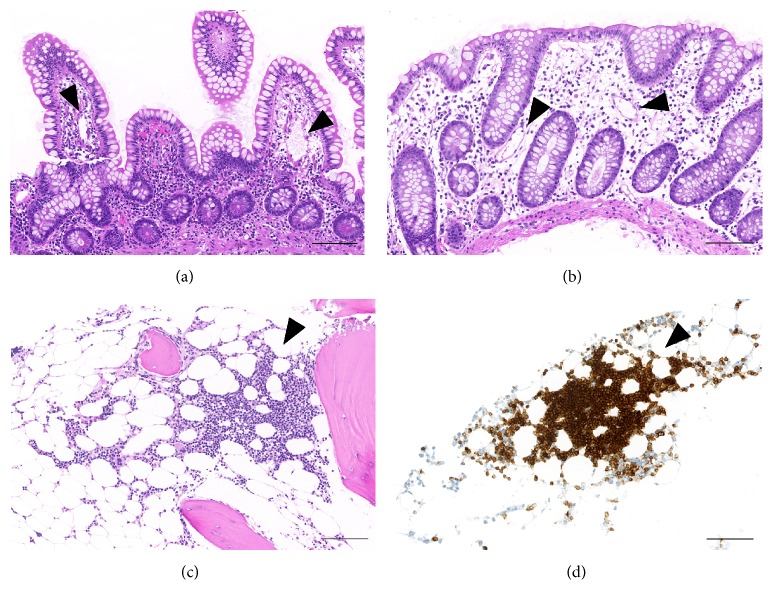
Small bowel (a) and large bowel (b) biopsy samples with minimal ectasia of lymphatic vessels (arrowheads) without other histopathologic findings (H&E). Bone marrow biopsy (c and d) with monotonous infiltrates of a low-grade non-Hodgkin lymphoma (c; H&E), highlighted by immunohistochemistry (d; B cell marker CD79a) (arrowheads). Scale bars = 100 *µ*m.

Repetitive upper and lower bowel endoscopy with biopsies were unremarkable without signs of Ménétrier's disease or lymphoma infiltration. Video capsule endoscopy and single-balloon enteroscopy revealed two small jejunal ulcerous lesions with unremarkable histology. Minimal lymphangiectasia was found on biopsies of the small and large intestine (Figure 1). Extensive work-up of all biopsies did not show pathogens, intraepithelial lymphocytosis, deposition of a paraprotein, or amyloidosis. Neither in abdominal wall fat pad biopsies amyloid deposits were found. Common variable immunodeficiency has been excluded after adequate immunologic response to vaccination with diphtheria/tetanus and pneumococci.

Sprue was ruled out by serological testing, HLA analysis, and intestinal biopsies.

Echocardiogram was unremarkable.

Serum protein electrophoresis was without paraproteinemia. Immunofixation demonstrated the presence of a weak IgM-kappa band. IgM in the serum was not elevated (0.7 g/L) and free-kappa light chains were slightly elevated in the serum and the urine (19.5 mg/L, norm 3.3–19.4; urine: 29.4 mg/L, norm <7.1). Bone marrow examination was diagnostic for low-grade B cell non-Hodgkin lymphoma with minor infiltration (20%) with predominant expression of IgG-*κ*. Genetic work-up showed monoclonal IgH rearrangement and mutated* MYD88* with a normal karyotype. We diagnosed marginal zone lymphoma although lymphoplasmacytic lymphoma formally cannot be excluded [13]. FDG-PET-scan showed increased metabolic activity along several large intestinal loops but no additional extranodal or nodal manifestations were found on FDG-PET- or CT-scan. A lymphography was normal.

**Figure 2:**
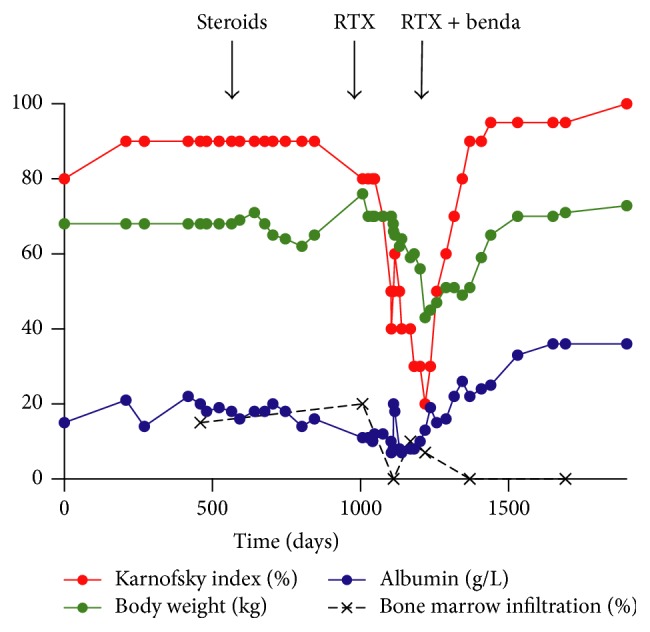
Evolution of representative disease markers. Courses of Karnofsky index, body weight, serum albumin, bone marrow infiltration, and timing of treatment are indicated. Please note different units of all markers (% for Karnofsky index and bone marrow infiltration, kg for body weight, and g/L for serum albumin). RTX: rituximab; benda: bendamustine.

Increasing symptoms prompted steroid treatment. Neither 6 cycles of dexamethasone (40 mg i.v. during 4 out of 28 days) nor oral prednisone (7.5 mg daily for 6 months) or budesonide (9 mg per day for 1 year) resulted in lasting improvement. Due to progressive symptoms with increasing edema and reduced quality of life accompanied by persistent minor bone marrow infiltration therapy with rituximab (4 cycles of 375 mg/m^2^ once weekly) was started. Even though this treatment temporarily improved diarrhea, patient deterioration and progressive weight loss of altogether 23 kg resulted in emergent inpatient admission (Figure 2).

The patient consumed a diet high in protein but low in long-chain fatty-acids but tolerated very little food. Feeding via a gastroduodenal tube resulted in worsening diarrhea and parenteral nutrition was started. A trial of octreotide (0.1 mg three times per day s.c. over 10 days) did not relieve symptoms. Progressive deterioration and hypotension prompted temporary treatment at an intensive-care unit.

A repeat bone marrow examination revealed minimal infiltration by the lymphoma, only detectable by flow cytometry. After exclusion of all alternative explanations for PLE, immunochemotherapy (rituximab 375 mg/m^2^ every 4 weeks, bendamustine 90 mg/m^2^ days 1 and 2 for a total of six months) was started. After one cycle, bone marrow examination showed complete response with improvement in performance, body weight, and edema.

20 months after completion of immunochemotherapy, lymphoma shows ongoing morphological, immunophenotypical, and molecular (MYD88 PCR) remission. The patient regained full performance status and body weight with normal serum albumin levels (Figure 2). The patient still notes occasional diarrhea which does not disturb daily activities. He provided written informed consent for publication of this case.

## 3. Discussion

We present the case of a patient with PLE secondary to marginal zone lymphoma. Protein losing enteropathy and intestinal malfunction resulted in pronounced clinical deterioration. Careful exclusion of virtually all alternative diagnosis and relief of PLE symptoms after treatment of lymphoma provide strong evidence of a causal relationship between marginal zone lymphoma and PLE in this patient.

Protein losing enteropathy was suspected due to the combination of low albumin and reduced serum levels of IgA, IgG, IgM, and transferrin combined with gastrointestinal symptoms. Proof of PLE was provided by determination of alpha-1 antitrypsin clearance which is excreted in the stool but not degraded [14, 15]. Normal values for alpha-1 antitrypsin clearance are 24 mL/day in patients without diarrhea and 56 mL/day in patients with diarrhea [16] and measurements in our patient were clearly above this level.

How B cell lymphoma caused PLE in our patient remains unclear but several potential mechanisms exist: (i) affection of intestinal lymph nodes resulting in lymphatic stasis, (ii) lymphatic hyperviscosity syndrome due to IgM paraprotein, (iii) epithelial deposition of the paraprotein, (iv) direct infiltration of the mucosa by lymphoma cells, (v) aberrant activity of the paraprotein, and (vi) paraneoplastic effect mediated by cytokines or by immune response to lymphoma.

In our patient an extensive work-up excluded several of the mechanisms described above. Imaging of lymph nodes was normal and none of the biopsies revealed pronounced lymphangiectasia. Concentrations of the paraprotein were consistently extremely low and no hyperviscosity syndrome was present. Epithelial deposition and mucosal infiltration were excluded by endoscopy with multiple biopsies and immunohistochemistry was negative for IgM deposition. Congo red staining was negative for amyloidosis in the intestinal mucosa and in abdominal fat. There was little association between extent of bone marrow infiltration by the lymphoma and severity of the patient's symptoms.

PLE has been described as a rare complication of B cell neoplasms. The largest case series summarizes 25 current and historical cases with Waldenstrom's macroglobulinemia and variable concentrations of IgM paraprotein [17]. In most patients intestinal lymphangiectasia was apparent on intestinal biopsies and immunohistochemistry demonstrated IgM deposition in intestinal lymphatic vessels. The authors speculate that mucosal IgM production would lead to accumulation of the IgM paraprotein in the intestinal lymphatic system and subsequent stasis.

Case reports demonstrated PLE in the setting of a grade II follicular non-Hodgkin lymphoma with pronounced duodenal B cell follicles and retroperitoneal lymph nodes [18] as well as a stage IVa non-Hodgkin lymphoma with retroperitoneal lymphadenopathy [19], respectively, with response to a treatment with rituximab CHOP. In another case of follicular lymphoma direct leakage of lymph from the duodenal bulb was observed [20].

Primary intestinal lymphangiectasia (PIL) is a rare condition [11] that can be complicated by the development of lymphoma [21, 22]. It remains unclear whether in these cases lymphangiectasia promotes lymphoma or an indolent lymphoma precedes lymphangiectasia. Reports of successful resolution of PIL-symptoms after lymphoma treatment [21] raise the possibility that some cases of PIL actually represent lymphoma associated PLE.

Treatment of PLE should focus on the underlying condition. Supportive treatment includes a low-fat, high-protein, medium triglyceride supplement diet [23] which has been ineffective in our patient. Octreotide has been used successfully in patients with primary intestinal lymphangiectasia [24], Ménétrier's disease [25], or amyloidosis [26] but did not affect symptoms in our patient. In our case even gastric or jejunal feeding and parenteral nutrition could not stop progressive deterioration. Finally, immunochemotherapy was started which resulted in a lasting remission of all symptoms.

Our case is remarkable for several reasons. (i) PLE was severe and almost lethal in our patient with an otherwise indolent lymphoma. (ii) Despite extensive investigations no macroscopic affection of the intestine was evident and intestinal lymphangiectasia was mild at best. Therefore, our case confirms that lymphoma can cause PLE without macroscopic lesions, without pronounced lymphangiectasia, and without enlarged intestinal lymph nodes. To the best of our knowledge, in all cases with lymphoma and PLE at least one of these pathologies had been present. (iii) The bone marrow was the only site where lymphoma was detectable. The lymphoma mass was low, suggesting that PLE was caused by humoral factors secreted by lymphoma cells (e.g., interleukins) or an immune response. (iv) To the best of our knowledge no PLE secondary to marginal zone lymphoma has been described previously.

Our case report has several limitations. (i) No surgical specimen and no biopsies of intestinal lymph nodes are available and infiltration of deeper lymphatic vessels by B cells remain possible. (ii) Pathological assessment of intestinal lymphangiectasia has not been standardized and our pathological assessment might differ from assessments in other case reports or case series. (iii) Limited conclusions can be drawn from a single case.

In summary, we report the case of a patient with severe protein losing enteropathy due to marginal zone lymphoma which was successfully treated with immunochemotherapy. Hidden B cell lymphoma should be considered if no cause of PLE is apparent after work-up of a patient. Due to lack of detectable intestinal lesions during endoscopic investigations and with most imaging techniques a high level of suspicion for lymphoma is warranted in all cases of unexplained PLE after standard evaluations.

## Figures and Tables

**Table 1:** Blood work at presentation in our unit.

	Value	Reference values
Hemoglobin (g/L)	163	134–170

White blood count (G/L)	9.2	3–9.6
Neutrophils	5.1	1.4–8
Monocytes	0.55	0.16–0.95
Eosinophils	0.35	0–0.7
Basophiles	0.05	0–0.15
Lymphocytes	3.3	1.5–4

Platelets (G/L)	332	143–400

Albumin (g/L)	18	40–49
Protein (g/L)	38	66–87

IgA (g/L)	0.4	0.7–4

IgG (g/L)	1.9	7–16

IgM (g/L)	0.7	0.4–2.3

Fibrinogen (g/L)	2.7	1.5–4

Transferrin (*µ*mol/L)	13	25–50

Chromogranin A (*µ*g/L)	1100	<85

Gastrin (ng/L)	345	13–115
